# Editorial: New Trends in Vascular Inflammation Research: From Biology to Therapy

**DOI:** 10.3389/fcvm.2019.00102

**Published:** 2019-08-28

**Authors:** Masanori Aikawa, Ichiro Manabe, Nikolaus Marx

**Affiliations:** ^1^Cardiovascular Division, Department of Medicine, Harvard Medical School, Brigham and Women's Hospital, Boston, MA, United States; ^2^Department of Disease Biology and Molecular Medicine, Chiba University, Chiba, Japan; ^3^Department of Internal Medicine I, Cardiology, University Hospital RWTH Aachen, Aachen, Germany

**Keywords:** inflammation, monocytes/macrophages, platelets, thrombosis, aging, vascular disease, atherosclerosis, vein graft disease

The evidence from basic science and clinical studies has established the role of inflammation in atherosclerosis and other vascular diseases. Many patients on potent drugs for modifiable risks, such as cholesterol-lowering statins and PCSK9 inhibitors, still suffer from vascular complications, including acute myocardial infarction. To tackle such residual risk, new medical therapies that more specifically target mechanisms for excessive inflammation may be needed. We believe that exploring novel mechanisms for vascular inflammation is a first stride toward the development of such new medical solutions. This Research Topic features 18 articles on new trends in vascular inflammation research with a focus on disease mechanisms (Part 1) on the one hand and new therapies (Part 2) on the other hand, all authored by leaders in vascular inflammation biology.

## Part I: Updates on the Mechanisms for Cardiovascular Inflammation (12 Articles)

Accumulating evidence suggests that monocytes and macrophages are heterogeneous and their subpopulations may have distinctive roles in vascular inflammation. Buscher et al. discuss monocyte heterogeneity with a focus on “patrolling” monocytes. Their article offers the emerging knowledge of the roles and kinetics of this monocyte subset as well as new technologies for identification and functional assays. Decano and Aikawa then provide the updates for macrophage biology in vascular disease. They focus on the mechanisms for activation, changes in intracellular metabolism, and current understanding of heterogeneity, and further discuss new paradigms of discovery science in vascular inflammation.

Thrombogenicity is a key feature of inflamed vessels, particularly in the diabetic milieu. Pechlivani and Ajjan discuss mechanisms for the imbalance of thrombotic and fibrinolytic factors, pathways responsible for increased thrombogenicity in diabetes, and therapeutic agents for thrombosis. This review emphasizes the importance of targeting diabetes-specific mechanisms for thrombosis.

While pro-inflammatory pathways may contribute to vascular disease, the impact of impaired protective mechanisms that support the hemostasis of non-diseased vessels also deserves similar levels of attention. An article by Yurdagul et al. discusses the role of defective efferocytosis of macrophages, a mechanism that clears apoptotic cells and promotes the resolution of inflammation, in the formation of necrotic core and the onset of acute thrombotic events. Miyazaki and Miyazaki then review the contribution of impaired protein catabolism to atherogenesis, focusing on the ubiquitin-proteasome pathway, autophagy, and the calpine system.

Many studies have reported the role of non-coding RNAs in cancer and neurologic disorders. More recently, we have learned that various non-coding RNAs contribute to cardiovascular diseases. In vascular biology, the evidence for the role of long non-coding RNAs, as compared to microRNAs, remains scant. Haemmig et al. overview how long non-coding RNAs promote vascular inflammation and future perspectives of this area.

Implantation of a autologous vein graft to bypass an obstructive coronary or peripheral artery is a common procedure. Rates for the occlusion or narrowing of vein grafts, however, are unacceptably high. Better understanding of underlying mechanisms will help to establish new therapies that prevent vein graft failure. An article by de Vries and Quax provides a comprehensive review of inflammatory mechanisms for the vein graft lesion development.

Members of the Krüppel-like factor (KLF) family of zinc-finger containing transcription factors regulate many biological processes. Accumulating evidence has implicated KLFs in cardiovascular biology. Two comprehensive reviews focus on two different contexts. Sweet et al. overview the role of KLFs in the biology of cell types related to vascular diseases (e.g., endothelial cells, smooth muscle cells, monocytes/macrophages), and strategies for pharmacologic modulations. Manabe and Oishi then discuss the biology of KLFs in key metabolic organs such as the liver and skeletal muscles and their disorders, and provide future perspectives.

Two articles review aging from different angles. Sanada et al. discuss cell senescence and dysregulation of innate immunity that contribute to chronic low-grade vascular inflammation in the elderly. Katsuumi et al. link cellular senescence with age-related disorders, such as heart failure, atherosclerotic vascular diseases, and metabolic syndrome.

At the end of Part I on the mechanisms for vascular disease, Yamazaki and Mukouyama review the role of pericytes in vascular disease with a specific emphasis on their heterogeneity.

## Part II: Emerging Evidence on New Therapies for Vascular Inflammation (6 articles)

This part covers a wide range of translational vascular medicine that spans from experimental validation of therapeutic targets to cardiovascular outcome trials. Sena et al. propose that cathepsin S is a potential therapeutic target for vascular inflammation and calcification. Peripheral artery disease is a global burden which shows an increasing prevalence and incidence worldwide. An original report by Nishimoto et al. demonstrates that activation of Toll-like receptor 9-mediated signaling by cell-free DNA released from ischemic tissues promotes macrophage activation and impairs blood flow recovery in the ischemic limb. An original report by Akita et al. demonstrates that the blockade of the IL-6 receptor suppresses atherogenesis in mice. Katsuki et al. then provides a comprehensive review of nanotechnology-based drug delivery and imaging for cardiovascular disease with a focus on inflammation. Rahman and Fisher provide a comprehensive review on the experimental and clinical evidence for the regression of atherosclerotic lesions and underlying mechanisms. They particularly focus on the role of macrophages. The last article by Aday and Ridker overviews the strong clinical evidence for the inflammatory aspects of atherosclerosis based on large cardiovascular outcome trials, including CANTOS that directly tested the effects of an anti-inflammatory therapy.

## Author Contributions

All authors listed have made a substantial, direct and intellectual contribution to the work, and approved it for publication.

### Conflict of Interest Statement

The authors declare that the research was conducted in the absence of any commercial or financial relationships that could be construed as a potential conflict of interest.

